# The Diamine Oxidase Gene Is Associated with Hypersensitivity Response to Non-Steroidal Anti-Inflammatory Drugs

**DOI:** 10.1371/journal.pone.0047571

**Published:** 2012-11-12

**Authors:** José A. G. Agúndez, Pedro Ayuso, José A. Cornejo-García, Miguel Blanca, María J. Torres, Inmaculada Doña, María Salas, Natalia Blanca-López, Gabriela Canto, Carmen Rondon, Paloma Campo, José J. Laguna, Javier Fernández, Carmen Martínez, Elena García-Martín

**Affiliations:** 1 Departamento de Farmacología, Universidad de Extremadura, Cáceres, Spain; 2 Departamento de Bioquímica y Biología Molecular y Genética, Universidad de Extremadura, Cáceres, Spain; 3 Laboratorio de Investigación, Fundación IMABIS, Málaga, Spain; 4 Servicio de Alergologia, Hospital Carlos Haya, Málaga, Spain; 5 Servicio de Alergologia, Hospital Infanta Leonor, Madrid, Spain; 6 Servicio de Alergologia, Hospital Cruz Roja, Madrid, Spain; 7 Servicio de Alergologia, Hospital San Juan, Alicante, Spain; French National Centre for Scientific Research, France

## Abstract

Non-steroidal anti-inflammatory drugs (NSAIDs) are the drugs most frequently involved in hypersensitivity drug reactions. Histamine is released in the allergic response to NSAIDs and is responsible for some of the clinical symptoms. The aim of this study is to analyze clinical association of functional polymorphisms in the genes coding for enzymes involved in histamine homeostasis with hypersensitivity response to NSAIDs. We studied a cohort of 442 unrelated Caucasian patients with hypersensitivity to NSAIDs. Patients who experienced three or more episodes with two or more different NSAIDs were included. If this requirement was not met diagnosis was established by challenge. A total of 414 healthy unrelated controls ethnically matched with patients and from the same geographic area were recruited. Analyses of the SNPs rs17740607, rs2073440, rs1801105, rs2052129, rs10156191, rs1049742 and rs1049793 in the *HDC*, *HNMT* and *DAO* genes were carried out by means of TaqMan assays. The detrimental DAO 16 Met allele (rs10156191), which causes decreased metabolic capacity, is overrepresented among patients with crossed-hypersensitivity to NSAIDs with an OR  = 1.7 (95% CI  = 1.3–2.1; Pc  = 0.0003) with a gene-dose effect (P = 0.0001). The association was replicated in two populations from different geographic areas (Pc  = 0.008 and Pc  = 0.004, respectively).

**Conclusions and implications:**

The *DAO* polymorphism rs10156191 which causes impaired metabolism of circulating histamine is associated with the clinical response in crossed-hypersensitivity to NSAIDs and could be used as a biomarker of response.

## Introduction

Drug-induced hypersensitivity reactions account for 5–10% of all adverse drug reactions, are generally dose independent, and involve specific immune or other mechanisms in which mediators are released after interaction between the drug and effectors [1]. The predisposing factors to hypersensitivity reactions with individual drugs are, in most cases, unknown. Hence, drug-induced hypersensitivity reactions are considered multigenic and multifactorial processes which depend on a complex interaction between genetic and environmental factors [2]. Environmental factors such as age, gender, alcohol intake, preexisting disorders and viral infections are relevant determinants in the development of drug-induced hypersensitivity reactions. Besides these factors, genetic susceptibility is a likely determinant in the severity and pattern of the clinical manifestations in these reactions [3]. Nevertheless, genetic factors underlying the risk of developing hypersensitivity to most drugs, and the presentation of clinical manifestations, have still to be fully understood.

Non-steroidal anti-inflammatory drugs (NSAIDs) have been reported to be the first most common cause of drug-related hypersensitivity reactions [4], [5]. Among the most common of these hypersensitivity reactions are cross-reactions involving chemically unrelated NSAIDs. These cross-reactions are believed to be triggered by non-specific immune mechanisms related to the arachidonic acid pathway and the COX-1 enzyme; these mechanisms, however, remain to be fully understood [4]. Independently of the triggering mechanisms, clinical symptoms of NSAIDs-induced hypersensitivity depend on the release of histamine and other mediators [4], [6]. The usefulness of oral antihistamine drugs in the symptomatic treatment of some drug hypersensitivity reactions is well documented [1], [4], [7], and therefore it could be hypothesized that functional polymorphisms of genes related to histamine homeostasis, including genes coding for enzymes involved in histamine synthesis and catabolism, could modify the clinical presentation or the timing of hypersensitivity reactions to NSAIDs [8]. In fact, in recent years, an association between polymorphisms of histamine metabolizing enzymes and clinical presentation of allergic diseases has been demonstrated [9], [10], [11], [12], [13].

Histamine biosynthesis is performed in one step, solely by the enzyme L-histidine decarboxylase (HDC, E.C. 4.1.1.22). Two enzymes are known to participate in the degradation of histamine: histamine N-metyltransferase (HNMT, E.C. 2.1.1.8) which is responsible for inactivating histamine in brain, and diamine oxidase (DAO; E.C. 1.4.3.6), responsible for scavenging extracellular histamine after mediator release [8]. The *HDC* gene is located in chromosome 15q21-q22. Two common nonsynonymous single nucleotide polymorphisms (SNPs) which give rise to the amino acid substitutions Thr31Met (rs17740607) and Glu644Asp (rs2073440) have been described in Caucasian individuals, the latter having clinical implications [13]. The *HNMT* gene is located in chromosome 2q22.1. One common nonsynonymous SNP causing Thr105Ile amino-acid substitution (rs1801105) is associated with reduced thermal stability and decreased HNMT activity [14], [15]. No other nonsynonymous SNPs with frequencies over 0.01 have been described for this gene [8]. The human *DAO* gene, which codes for the DAO enzyme, is located in chromosome 7q34-q36. Three common nonsynonymous SNPs, which bring about the amino acid substitutions Thr16Met (rs10156191), Ser332Phe (rs1049742) and His645Asp (rs1049793) have been identified in Caucasian individuals, and the functional effects of these SNPs in enzyme activity have been studied in detail [16], [17]. Recently an additional promoter, SNP rs2052129 (G4586T), which seems to cause decreased transcriptional activity, has been described [11].

With the aim of disclosing mechanisms located downstream of the triggering factors of NSAIDs-induced hypersensitivity, which may influence the clinical presentation of these common adverse drug reactions, we analyzed functional polymorphisms in genes related to histamine homeostasis in a large group of well-phenotyped patients suffering from crossed-hypersensitivity to NSAIDs.

## Patients and Methods

We studied a cohort of 442 unrelated Caucasian patients with hypersensitivity to NSAIDs. Table 1 summarizes the clinical characteristics of the subjects in the study groups. Only patients who had experienced more than two episodes with two or more different NSAIDs, classified as cross-reactivity, were included in the study. Of these, 342 were recruited from the Allergy Service, Carlos Haya Hospital (Málaga, Spain) and 100 were recruited from the Allergy Service, Infanta Leonor Hospital (Madrid, Spain), and from the Cruz-Roja General Hospital (Madrid, Spain). These subgroups were selected in separate hospitals in order to analyze whether putative associations are replicated in geographically independent populations. The patients were assessed in each of the allergy departments of the corresponding clinical centres by an allergy specialist. The diagnostic criteria used for hypersensitivity to NSAIDs are described elsewhere [18]. Atopy status was assessed in both patients and controls by means of three parameters: clinical questionnaire, skin prick test and measurement of total IgE in serum samples. In those cases with cutaneous involvement where the diagnosis was not confirmed, a drug provocation test was carried out as described elsewhere [18]. This applies to those cases with less than three episodes. Those with three or more episodes with at least two different NSAIDs were considered positive as has been demonstrated by Blanca-López et al., Clin Exp Allergy, 2012, in press. In all patients with respiratory symptoms diagnosis was confirmed by nasal provocation test [19]. From the total number of cases included (n = 442), the 15% had respiratory involvement and positive provocation test, as required in the inclusion criteria. In the remaining groups, where cutaneous involvement was the common characteristic, in 40% the diagnosis was confirmed by challenge as they had suffered only one or two episodes. For 60% diagnosis was established on the basis of three or more episodes of at least two different drugs. Clinical characterisation included questionnaires on the drug responsible for the hypersensitivity reaction, other pharmacological therapy, allergic diseases, familial antecedents (defined as the presence of disease in a first or second degree relative), age, gender, determination of total and specific IgE, haemogram with determination of eosinophils, skin tests for common food or environmental allergens, pulmonary function tests, and smoking and drinking habits. Patients were classified into 3 groups according to their clinical symptoms: urticaria and angioedema; asthma and/or rhinitis (respiratory symptoms) and anaphylaxis. Four hundred and fourteen healthy unrelated control subjects, ethnically matched with patients and from the same geographic area, were recruited: 268 in Málaga and 146 in Madrid. All individuals were Caucasians. Control subjects were selected from medical students and the staff of the universities and the hospitals participating in the study. Medical examinations were performed and histories obtained from each individual to exclude pre-existing disorders. All control subjects reported previous consumption of NSAIDs with no adverse effects.

**Table 1:** Clinical characteristics of the study subjects.

	Málaga	Málaga	Madrid	Madrid
	Patients (n = 342)	Healthy subjects (n = 268)	Patients (n = 100)	Healthy subjects (n = 146)
**Women, n (%)**	215 (62.9%)	165 (61.6%)	56 (56.0%)	92 (63.0%)
**Age ± sd (range)**	41.0 ± 15.2 (5–92)	38.9±15.2 (15–79)	44.3±16.3 (14–89)	44.6±12.1 (18–77)
**Antecedents of atopy**	80 (23.4%	12 (6.7%)	3 (3.0%)	0
**Interval <1 h; n (%)**	174 (50.9%)	n.a.	53 (53.0%)	n.a.
**Interval 1**–**6 h; n (%)**	66 (19.3%)	n.a.	10 (10.0%)	n.a.
**Interval 6**–**24 h; n (%)**	9 (2.6%)	n.a.	2 (2.0%)	n.a.
**Interval >24 h; n (%)**	5 1.5%)	n.a.	1 (1.0%)	n.a.
**Interval unknown**	88 (25.7%)	n.a.	34 (34.0%)	n.a.
**Urticaria+ Angiooedema**	228 (66.6%)	n.a.	31 (31.0%)	n.a.
**Anaphylaxis**	20 (5.8%)	n.a.	3 (3.0%)	n.a.
**Respiratory symptoms**	31 (9.1%)	n.a.	36 (36.0%)	n.a.
**Mixed symptoms**	63 (18.4%)	n.a.	18 (18.0%)	n.a.
**Other symptoms**	0	n.a.	12 (12.0%)	n.a.
**Total IgE (IU/mL) ± sd (range)**	130.11±180.63 (2.16–1485)	70.91±157.78 (0–1510)	not determined	not determined
**Causative drugs**				
**Ibuprofen**	210	n.a.	57	n.a.
**Aspirin**	194	n.a.	64	n.a.
**Metamizole**	125	n.a.	39	n.a.
**Diclofenac**	73	n.a.	21	n.a.
**Acetaminophen**	57	n.a.	11	n.a.
**Dexketoprofen**	18	n.a.	10	n.a.
**Naproxen**	17	n.a.	6	n.a.
**Piroxicam**	12	n.a.	1	n.a.
**Propiphenazone**	3	n.a.	3	n.a.
**Ketorolac**	11	n.a.	1	n.a.
**Ketoprofen**	6	n.a.	3	n.a.
**Lysine clonixinate**	7	n.a.	1	n.a.
**Indometacin**	4	n.a.	2	n.a.
**Etoricoxib**	4	n.a.	0	n.a.
**Aceclofenac**	2	n.a.	1	n.a.
**Meloxicam**	2	n.a.	1	n.a.
**Lornoxicam**	3	n.a.	0	n.a.
**Piketoprofen**	2	n.a.	0	n.a.
**Aminophenazone**	1	n.a.	0	n.a.
**Etofenomate**	1	n.a.	0	n.a.
**Phenylbutazone**	1	n.a.	0	n.a.
**Celecoxib**	1	n.a.	0	n.a.

Many patients reacted to two or more drugs.

### Ethics Statement

All the patients and over 95% of the healthy subjects requested to participate in the study agreed to do so. All participants gave written consent for their participation. The protocol complied with the Declaration of Helsinki and its subsequent revisions and was approved by the Ethics Committees of the Carlos Haya Hospital, Málaga, the Infanta Leonor Hospital, Madrid, and the University of Extremadura, Badajoz, Spain.

### Genotype analysis

Genomic DNA was obtained from peripheral leukocytes and purified in accordance with standard procedures. The SNPs analyzed were selected according to allele frequencies (over 0.01) in the population study, and either functional or clinical relevance, in accordance with published evidence [8], [9], [10], [11], [14], [16]. Apart from the SNPs analyzed in this study, no additional nonsynonymous SNPs occur in Caucasian subjects with a significant allele frequency in the genes analyzed according to public databases (http://browser.1000genomes.org). Genotyping was performed by means of TaqMan assays (Applied Biosciences Hispania, Alcobendas, Madrid, Spain) designed to detect the following SNPs: HNMT: rs11558538 (C__11650812_20); DAO: rs2052129 (C__11630976_1), rs10156191 (C__25593951_10), rs1049742 (C___7599782_20, rs1049793 (C___7599774_10); HDC: rs17740607 (C__25624415_20) and rs2073440 (C__15950871_20). Detection was carried out by qPCR in an Eppendorf realplex thermocycler using fluorescent probes. The amplification conditions were as follows: after a denaturation time of 10 min at 96°C, 45 cycles of 92°C 15 sec 60°C 90 sec were carried out and fluorescence was measured at the end of each cycle and at endpoint. All samples were determined in triplicate and genotypes were assigned both by means of gene identification software (RealPlex 2.0, Eppendorf) and by analysis of the reference cycle number for each fluorescence curve, calculated using the CalQPlex algorithm (Eppendorf).

### Statistical analyses

Hardy-Weinberg equilibrium (HWE), allelic and genotype analyses were performed with PLINK software [20]. Haplotype reconstruction was performed using the program PHASE v2.1.1 [21]. We used the default model for recombination rate variation with 1000 iterations, 500 burn-in iterations and a thinning interval of 1. Diplotypes were obtained from the combination of haplotypes in the best run (the one that showed the maximum consistency of results across all runs); further details for haplotype disambiguation are provided elsewhere [22].

Statistical analyses were performed using SPSS 15.0 for Windows (SPSS Inc., Chicago, Illinois, USA). Intergroup comparison values and 95% confidence limits were calculated by Chi-square or Fisher tests when appropriate. Correction for multiple testing (Pc values) was performed according to Bonferroni's method. Differences were considered to be significant when Pc values were under 0.05.

The statistical power of the study sample size was evaluated with a genetic model analyzing the frequency for carriers of the disease gene with an OR value  = 2 (α = 0.05). With the sample size analyzed in this study the statistical power for two-sided and one-sided associations, respectively, for the risk related to the presence of the variant alleles was as follows: HNMT rs1801105, 98.4% and 99.3%; DAO rs2052129 100.0% and 100.0%, DAO rs10156191, 99.9% and 99.9%; DAO rs1049742, 94.0% and 96.9%; ABP rs1049793, 100.0% and 100.0%; HDC rs17740607 100.0% and 100.0%; HDC rs2073440 99.9% and 100%. After the genetic analysis, the power of the study was recalculated with the observed OR value for the SNP rs10156191. Two-sided and one-sided associations with risk adjusted to an OR value  = 1.7 (α = 0.05) were 98.4% and 96.7%, respectively, for carriers of the risk allele.

## Results

Genotype data were adjusted to dominant, recessive and allelic models. The best fit was obtained with the recessive model for the major allele and this model was used to calculate the P and the OR values. Table 2 shows the genotypes and the allelic variant frequencies for genes coding for histamine-metabolising enzymes (*HNMT* and *DAO*) among patients who developed hypersensitivity to NSAIDs and control subjects. A prominent difference, with a higher frequency for C/T and T/T *DAO* 16 Thr/Met genotypes among patients as compared to control subjects was observed. The high statistical significance of the comparisons shown in Table 2 remains after Bonferroni's corrections (*Pc* values). Armitage's test for trend indicates that the presence of the *DAO* 16 Met allele increases the risk with a gene-dose effect (*P* = 0.0001). Besides the *DAO* 16 Thr/Met polymorphism, no major differences in genotypes or allele frequencies were observed in other SNPs related to histamine-metabolising enzymes. *HDC* genotypes and allele frequencies are shown in Table 3. Frequency of the Glu644 allele was significantly higher in the patients than in the control group, although the statistical significance of the *Pc* values is weak.

**Table 2:** Genotypes of genes coding for the histamine-metabolising enzymes HNMT and DAO in the study group.

SNP	Subjects	No.of subjects	Genotype Frequencies %			p	OR(95% CI)	Allele Frequencies,%		p	OR (95% CI)
**rs1801105 HNMT Thr105Ile**			C/C	C/T	T/T			C	T		
	Patients	442	85.5	14.0	0.5	0.214	0.88 (0.60–1.27)	92.5	7.5	0.350	0.85 (0.60–1.20)
	Controls	414	83.8	15.0	1.2	reference		91.3	8.7		reference
**rs2052129 DAO Promoter**			G/G	G/T	T/T			G	T		
	Patients	442	54.8	38.2	7.0	0.587	1.08 (0.82–1.42)	73.9	26.1	0.587	1.06 (0.85–1.32)
	Controls	414	52.9	39.6	7.5	reference		72.7	27.3		reference
**rs10156191 DAO Thr16Met**			C/C	C/T	T/T			C	T		
	Patients	442	53.8	37.1	9.0	0.0002	1.68 (1.27–2.12)	72.4	27.6	0.00006 Pc = 0.0003	1.59 (1.27–2.00)
	Controls	414	66.2	29.0	4.8	reference		80.7	19.3		reference
**rs1049742 DAO Ser332Phe**			C/C	C/T	T/T			C	T		
	Patients	442	86.9	12.0	1.1	0.630	0.91 (0.62–1.34)	92.9	7.1	0.774	0.95 (0.66–1.37)
	Controls	414	85.7	13.5	0.7	reference		92.5	7.5		reference
**rs1049793 DAO His645Asp**			C/C	C/G	G/G			C	G		
	Patients	442	53.6	38.5	7.9	0.778	0.96 (0.73–1.26)	72.9	27.1	0.902	0.99 (0.80–1.22)
	Controls	414	52.7	39.9	7.5	reference		72.6	27.4		reference

**Table 3:** *HDC* genotypes in the study group.

SNP	Subjects	No.of subjects	Genotype Frequencies %			p	OR (95% CI)	Allele Frequencies,%		p	OR (95% CI)
**rs17740607 Thr31Met**			A/A	A/G	G/G			A	G		
	Patients	442	83.5	15.4	1.1	0.698	1.29 (0.39–4.24)	91.2	8.8	0.934	1.01 (0.73–1.42)
	Controls	414	83.6	15.0	1.4	reference		91.1	8.9		reference
											
**rs2073440 Glu644Asp**			A/A	A/C	C/C			A	C		
	Patients	442	93.7	6.1	0.2	0.525	2.14 (0.19–23.70)	96.7	3.3	0.009 Pc = 0.018	1.85 (1.16–2.97)
	Controls	414	88.6	10.9	0.5	reference		94.1	5.9		reference

To ensure reproducibility and to confirm statistically significant associations, the two polymorphisms which showed a positive relationship (*DAO* Thr16Met and *HDC* Glu644Asp) were analyzed according to the origin of the participants: Malaga or Madrid. In the case of *HDC* Glu644Asp, the association could not be replicated: this polymorphism did not show statistically significant differences between both frequencies, genotype and allele, in the population subgroup from Madrid (Table S1). In contrast, the genotypic frequency for the *DAO* Thr16Met polymorphism was replicated as it was significantly different in both populations, Malaga and Madrid, compared to the control subjects (Table 4).

**Table 4:** *DAO rs10156191* genotypes in the two study subgroups.

SNP	Subjects	No.of subjects	Genotype Frequencies %			p	OR (95% CI)	Allele Frequencies,%		p	OR (95% CI)
**Discovery (Malaga, Spain)**			C/C	C/T	T/T			C	T		
	Patients	342	55.0	36.5	8.5	0.001	1.54 (1.11–2.14)	73.2	26.8	0.004 Pc = 0.008	1.48 (1.13–1.94)
	Controls	268	65.3	29.9	4.9	reference		80.2	19.8		reference
**Replication (Madrid, Spain)**			C/C	C/T	T/T			C	T		
	Patients	100	50.0	39.0	11.0	0.005	2.11 (1.25–3.56)	69.5	30.5	0.002 Pc = 0.004	1.93 (1.27–2.95)
	Controls	146	66.2	29.0	4.8	reference		80.7	19.3		reference

It has been shown that DAO activity depends on determined mutated haplotypes rather than isolated point mutations [16]. (Figure S1 shows the location of the *DAO* SNPs and a linkage plot for these SNPs in the population study). In particular, the alleles encoding for the 16 Met or the 645 Asp-containing proteins, either isolated or combined, cause impairment on enzyme activity. Table 5 shows the frequencies for *DAO* diplotypes, that is, the combination of phased haplotypes. All haplotypes containing the *DAO* Thr16Met were more frequent in patients than in control subjects. This indicates that the findings regarding *DAO* diplotypes in Table 2 are solely due to the occurrence of the *DAO* 16 Met allele rather than to the occurrence of allozymes with a particular combination of variant amino-acids besides 16 Met. (Table S2 shows a comparison of full *DAO* haplotype frequencies in patients with NSAID-hypersensitivity and control subjects).

**Table 5:** *DAO rs10156191* plus *rs1049793* haplotypes in the two study subgroups.

SNP	Subjects	No.of subjects	Diplotype Frequencies %			p	OR (95% CI)	Allele Frequencies,%		p	OR (95% CI)
**Discovery (Malaga, Spain)**			N/N	N/C	C/C			N	C		
	Patients	342	78.4	20.5	1.2	0.025	1.62 (1.06–2.48)	88.6	11.4	0.021 Pc = 0.042	1.60 (1.07–2.38)
	Controls	268	85.4	14.2	0.4	reference		92.5	7.5		reference
**Replication (Madrid, Spain)**			N/N	N/C	C/C			N	C		
	Patients	100	71.0	26.0	3.0	0.005	2.43 (1.29–4.58)	84.0	16.0	0.002 Pc = 0.004	2.46 (1.37–4.40)
	Controls	146	85.6	14.4	0.0	reference		92.8	7.2		reference
											
**Combined findings**			N/N	N/C	C/C			N	C		
	Patients	442	76.7	21.7	1.6	0.001	1.79 (1.26–2.55)	87.6	12.4	0.0005 Pc = 0.001	1.79 (1.29–2.48)
	Controls	414	85.5	14.3	0.2	reference		92.6	7.4		reference

N: non-carrier of the risk haplotype; C: Carrier of the risk haplotype.

The influence of confounders in the association of *DAO* Thr16Met and the risk of developing NSAID-hypersensitivity were analyzed in multivariate analyses. These confounders included the type of causing drug, gender, the presence of previous history of atopy or urticaria and the positivity of prick tests. No statistically significant differences among subgroups of patients were observed regarding these parameters (Table S3 shows the *DAO* Thr16Met genotype and allele frequencies regarding the type of drug triggering the hypersensitivity reaction). Table 6 shows the genotype and allele frequencies according to the clinical presentation of the hypersensitivity event. The association of *DAO* Thr16Met and the risk of developing NSAID-hypersensitivity was statistically significant for all clinical presentations, although the most significant association was observed in patients with respiratory symptoms.

**Table 6:** *DAO* Thr16Met genotypes according to clinical presentation.

SNP	No.of subjects	Genotype Frequencies %		p		OR (95% CI)	Allele Frequencies,%		p	OR (95% CI)
		C/C	C/T	T/T			C	T		
**Urticaria+ Angiooedema**	259	55.2	36.7	8.1	0.004	1.59 (1.16–2.18)	73.6	26.4	0.002 Pc = 0.008	1.50 (1.16–1.95)
		C/C	C/T	T/T			C	T		
**Anaphylaxis**	23	47.8	39.1	13.0	0.072	2.14 (0.92–4.96)	67.4	32.6	0.028 Pc = 0.112	2.02 (1.07–3.83)
		C/C	C/T	T/T			C	T		
**Respiratory symptoms**	67	46.3	40.3	13.4	0.002	2.27 (1.35–3.83)	66.4	33.6	0.0002 Pc = 0.0008	2.11 (1.42–3.14)
		C/C	C/T	T/T			C	T		
**Mixed symptoms**	81	53.1	39.5	7.4	0.025	1.73 (1.07–2.80)	72.8	27.2	0.024 Pc = 0.096	1.56 (1.06–2.29)

Twelve patients had other symptoms (See Table 1).

Since it has been shown that asthma and rhinitis patients who have variant *DAO* genotypes display lower IgE levels as compared with patients with no mutations [9], the distribution of IgE levels among patients with different risk genotypes was analyzed. The IgE levels, expressed as IU/mL were equal to 130.11±180.63 for patients and 70.91±157.78 for control subjects (*P*<0.001). No statistically significant differences were observed when patients were grouped according to *DAO* genotypes, thus indicating that the risk SNP identified in this study does not co-segregate with IgE levels.

## Discussion and Conclusions

Unpredictable adverse drug reactions related to NSAIDs are common in clinical practice. In recent years there has been an increasing interest in developing clinical models to allow more personalized NSAID therapy, but these models primarily address cardiovascular or gastrointestinal risks. In contrast, mechanisms underlying hypersensitivity reactions to NSAIDs have received less attention. It is intriguing that chemically unrelated NSAIDs and NSAIDs with different pharmacological mechanisms cause cross-hypersensitivity. Data accumulated so far suggest the involvement of peptidyl-leukotrienes and other mediators including histamine, as factors implicated in the clinical development of these hypersensitivity responses. [6], [23]. The triggering mechanisms for these crossed-reactions, although believed to be related to their pharmacological effects on the COX-1 enzyme, remain to be fully understood. Rather than the identification of risk factors related to the triggering of NSAIDs-induced hypersensitivity, the aim of this study is the identification of genetic factors related to events located downstream of the triggering reaction which, in fact, are the immediate determinants of the clinical presentation. Since we aimed to investigate a downstream event which is common regardless of drug-specific triggering mechanisms, we selected patients with cross-hypersensitivity rather than hypersensitivity to only one NSAID.

Although it is known that genetic factors play a role in hypersensitivity response to NSAIDs [24], that histamine has a prominent role in the hypersensitivity response to NSAIDs [6], and that functional polymorphisms exist which modulate histamine homeostasis [8], this is the first study to analyze a putative association between polymorphisms in genes coding histamine-metabolizing enzymes and the clinical presentation of hypersensitivity to NSAIDs. In this study we analyze the effect of functional SNPs in genes coding histamine synthesis and histamine-metabolizing enzymes on the clinical presentation of NSAIDs-induced hypersensitivity reactions. Strengths of our study include the involvement of a large number of well-phenotyped patients with hypersensitivity to different NSAIDs and different types of clinical response. In addition, the patients who participated in this study were recruited from several hospitals in two different geographic areas. This permitted comparison of different populations and replication of the genetic association study.

The present study shows a significant over-representation of the *DAO* 16Met polymorphism (rs10156191) in two populations of patients with hypersensitivity to NSAIDs. The presence of nonsynonymous SNPs in the *DAO* gene is a determinant of enzyme activity [16]. Specifically, the 16Met variant results in a reduction of serum DAO activity [16]. A genetically-determined decreased ability to metabolize extracellular histamine is a plausible mechanism to explain the increased frequency of this variant allele in individuals who develop clinical symptoms of hypersensitivity to NSAIDs. A recent study has shown that an additional *DAO* gene variant rs2052129, located in the gene promoter, has a significant effect on transcriptional activity and hence on DAO activity [11]. We did not find any association between the promoter SNP rs2052129 and the risk of developing NSAIDs hypersensitivity.

Since it has been shown that the combination of amino-acid exchanges 645Asp and 16Met in the DAO enzyme has a greater functional effect on the enzyme activity than the amino-acid exchange 16 Met alone [16], we analyzed the effect of this double-mutated haplotype on the risk of cross reactivity to NSAIDs. The results did not show a greater risk among carriers of the combined 16 Met +645Asp haplotype and carriers of the 16 Met SNP in isolation, thus indicating that the variant 16 Met determines the risk genotype. This tallies with the fact that the 16 Met is the SNP which caused the greatest decrease in enzyme activity among the nonsynonymous DAO SNPs analyzed [11], [16]. The risk genotype is not limited to a particular pattern of clinical manifestation (Table 6), although the frequency is higher among patients who develop respiratory symptoms. In agreement with the proposed mechanism downstream of the triggering factor of the reaction, no specific association of the DAO 16 Met allele with hypersensitivity triggered by a particular NSAID was observed in the study group (Table S3).

With regard to the *HDC* gene, we analyzed the frequency of two nonsynonymous SNPs in relation to their possible association with NSAID hypersensitivity. Although we detected differences in allele frequencies of Glu644Asp *HDC* between patients and control individuals, these differences were not replicated when we analyzed the two populations (Málaga and Madrid) separately, and therefore we cannot conclude that there is an unambiguous association between this genotype and the risk of hypersensitivity to NSAIDs. For the other HDC SNP studied, Thr31Met, the differences in genotypic and allelic frequencies among patients and control individuals were not significant. These results are in agreement with current knowledge on the effect of these polymorphisms on the function of the enzyme [8], [13].

To date, only one study on the association between polymorphisms of *HNMT* gene and hypersensitivity to NSAIDs has been carried out. This study is restricted to aspirin intolerant chronic urticaria in a Korean population [25]. Kim et al described an association between the development of chronic urticaria in patients intolerant to aspirin and the occurrence of the 939A> C polymorphism, which seems to decrease enzyme activity by reducing the stability of its mRNA, whereas the *HNMT* polymorphism 314C> T, which unambiguously causes a major impairment in enzymatic activity, did not show any association. Our study rules out any major association of the *HNMT* 314C> T polymorphism with the risk of developing NSAID-induced drug hypersensitivity.

In conclusion, the findings described in this study indicate that a detrimental mutation in the *DAO* gene is related to a clinical development of hypersensitivity to NSAIDs. These findings agree with the general model in which mutations in genes encoding histamine-metabolizing enzymes may increase the risk, or modify the clinical presentation, of allergic diseases in which histamine plays an important role. This finding could guide future research in the area of drug hypersensitivity as it points to a common mechanism which may affect the clinical presentation of hypersensitivity reactions, regardless of drug-specific triggering mechanisms.

## Supporting Information

Figure S1 **Scheme and linkage analysis of the** ***DAO*** **SNPs analyzed in this study.** Shaded areas correspond to the exons of the *DAO* gene. Four SNPs were analyzed for linkage disequilibrium in 414 unrelated healthy individuals (828 genes). Top of the Figure: for every SNP the position in chromosome 7 (contig NT_007914.15), the base change, the SNP identification and the minor allele frequencies (MAF) are shown. The linkage figure located at the bottom was composed with Haploview 4.1 according to the standard colour scheme (D'/LOD), and the D' values (× 100) are shown.
(DOCX)

Table S1 ***HDC rs2073440*** **genotypes in two subgroups of patients.**
(DOCX)

Table S2 **Comparison of** ***DAO*** **haplotype frequencies in patients with NSAID-hypersensitivity and control subjects.**
(DOCX)

Table S3 ***DAO*** **Thr16Met genotype and allele frequencies regarding the type of drug triggering the hypersensitivity reaction.**
(DOCX)
