# Supplementary material for: The Diamine Oxidase Gene Is Associated with Hypersensitivity Response to Non-Steroidal Anti-Inflammatory Drugs
Source: PLoS One. 2012 Nov 12;7(11):e47571. doi: 10.1371/journal.pone.0047571 (PMC3495953; doi:10.1371/journal.pone.0047571)
Supplement: Figure S1 — Scheme and linkage analysis of the DAO SNPs analyzed in this study. Shaded areas correspond to the exons of the DAO gene. Four SNPs were analyzed for linkage disequilibrium in 414 unrelated healthy individuals (828 genes). Top of the Figure: for every SNP the position in chromosome 7 (contig NT_007914.15), the base change, the SNP identification and the minor allele frequencies (MAF) are shown. The linkage figure located at the bottom was composed with Haploview 4.1 according to the standard colour scheme (D'/LOD), and the D' values (× 100) are shown. (DOCX) [file pone.0047571.s001.docx]

Figure S1. Scheme and linkage analysis of the *DAO* SNPs analyzed in this study.
