# Supplementary material for: The Diamine Oxidase Gene Is Associated with Hypersensitivity Response to Non-Steroidal Anti-Inflammatory Drugs
Source: PLoS One. 2012 Nov 12;7(11):e47571. doi: 10.1371/journal.pone.0047571 (PMC3495953; doi:10.1371/journal.pone.0047571)
Supplement: Table S1 — HDC rs2073440 genotypes in two subgroups of patients. (DOCX) [file pone.0047571.s002.docx]

Table S1. *HDC rs2073440* genotypes in two subgroups of patients.

| SNP | Subjects | No.of subjects | Genotype  Frequencies % | | | p | OR  (95% CI) | Allele  Frequencies,% | | p | OR (95% CI) |
| --- | --- | --- | --- | --- | --- | --- | --- | --- | --- | --- | --- |
| Discovery (Malaga, Spain) |  |  | A/A | A/C | C/C |  |  | A | C |  |  |
|  | Patients | 342 | 94.2 | 5.6 | 0.3 | 0.862 | 1.28 (0.08-20.51) | 96.9 | 3.1 | 0.014 | 2.01 (1.14-3.52) |
|  | Controls | 268 | 88.4 | 11.2 | 0.4 | reference |  | 94.0 | 6.0 |  | reference |
|  |  |  |  |  |  |  |  |  |  |  |  |
| Replication  (Madrid, Spain) |  |  | A/A | A/C | C/C |  |  | A | C |  |  |
|  | Patients | 100 | 92.0 | 8.0 | 0.0 | 0.407 | 2.07 (0.08-51.38) | 96.0 | 4.0 | 0.366 | 1.48 (0.63-3.51) |
|  | Controls | 146 | 89.0 | 10.3 | 0.7 | reference |  | 94.2 | 5.8 |  | reference |
