# Supplementary material for: The Diamine Oxidase Gene Is Associated with Hypersensitivity Response to Non-Steroidal Anti-Inflammatory Drugs
Source: PLoS One. 2012 Nov 12;7(11):e47571. doi: 10.1371/journal.pone.0047571 (PMC3495953; doi:10.1371/journal.pone.0047571)
Supplement: Table S2 — Comparison of DAO haplotype frequencies in patients with NSAID-hypersensitivity and control subjects. (DOCX) [file pone.0047571.s003.docx]

Table S2. Comparison of *DAO* haplotype frequencies in patients with NSAID-hypersensitivity and control subjects.

| Haplotype  (rs2052129-rs10156191-rs1049742-rs1049793) | Frequency cases (%) | Frequency controls (%) | Chi-Square | P |
| --- | --- | --- | --- | --- |
| G-C-C-C (non-mutated) | 51.6 | 51.5 | 0.00 | 0.973 |
| G-C-C-G | 14.8 | 17.0 | 1.63 | 0.201 |
| T-T-C-C | 12.5 | 10.4 | 2.08 | 0.149 |
| T-C-C-C | 5.3 | 8.4 | 6.72 | **0.009** |
| T-T-T-G | 4.7 | 3.2 | 2.66 | 0.103 |
| G-T-C-C | 4.5 | 4.2 | 0.135 | 0.713 |
| G-T-C-G | 3.2 | 1.0 | 10.24 | **0.001** |
| T-T-C-G | 2.3 | 1.5 | 1.233 | 0.267 |
| T-C-T-G | 1.2 | 2.8 | 6.325 | 0.012 |
