# Supplementary material for: The Diamine Oxidase Gene Is Associated with Hypersensitivity Response to Non-Steroidal Anti-Inflammatory Drugs
Source: PLoS One. 2012 Nov 12;7(11):e47571. doi: 10.1371/journal.pone.0047571 (PMC3495953; doi:10.1371/journal.pone.0047571)
Supplement: Table S3 — DAO Thr16Met genotype and allele frequencies regarding the type of drug triggering the hypersensitivity reaction. (DOCX) [file pone.0047571.s004.docx]

Table S3. *DAO* Thr16Met genotype and allele frequencies regarding the type of drug triggering the hypersensitivity reaction

| Genotype rs10156191  DAO Thr16Met | Overall patients:  N (%; 95% CI) | Ibuprofen  N (%; 95% CI) | AAS  N (%; 95% CI) | Metamizole  N (%; 95% CI) | Diclofenac  N (%; 95% CI) | Acetaminophen  N (%; 95% CI) |
| --- | --- | --- | --- | --- | --- | --- |
| Thr/Thr | 238 (53.8; 49.2-58.5) | 149 (55.8; 49.8-61.8) | 151 (58.5; 52.5-64.5) | 96 (58.5; 51.0-66.1) | 55 (58.5; 48.6-68.5) | 39 (57.4; 45.6-69.1) |
| Thr/Met | 164 (37.1; 32.6-41.6) | 98 (36.7; 30.9-42.5) | 88 (34.1; 28.3-39.9) | 53 (32.3; 25.2-39.5) | 32 (34.0; 24.5-43.6) | 24 (35.3; 23.9-46.7) |
| Met/Met | 40 (9.0; 6.4-11.7) | 20 (7.5; 4.3-10.6) | 19 (7.4; 4.2-10.6) | 15 (9.1; 4.7-13.6) | 7 (7.4; 2.1-12.8) | 5 (7.4; 1.1-13.6) |
| Alleles |  |  |  |  |  |  |
| Thr | 640 (72.4; 69.5-75.3) | 396 (74.2; 70.4-77.9) | 390 (75.6; 71.9-79.3) | 245 (74.7; 70.0-79.4) | 142 (75.5; 69.4-81.7) | 102 (75.0; 67.7-82.3) |
| Met | 244 (27.6; 24.7-30.5) | 138 (25.8; 22.1-29.6) | 126 (24.4; 20.7-28.1) | 83 (25.3; 20.6-30.0) | 46 (24.5; 18.3-30.6) | 34 (25.0; 17.7-32.3) |
